# Melanocytic nevi in sentinel lymph nodes: association with cutaneous nevi and clinical relevance in patients with cutaneous melanomas

**DOI:** 10.1007/s00432-021-03894-y

**Published:** 2022-01-20

**Authors:** Lutz Kretschmer, Viktor Schnabel, Christian Kromer, Christoph Bauer-Büntzel, Annika Richter, Felix Bremmer, Fabian Kück, Katharina Julius, Christina Mitteldorf, Michael P. Schön

**Affiliations:** 1 Department of Dermatology, Venereology and Allergology, University Medical Center, Robert Koch Str. 40, 37075 Göttingen, Germany; 2 Department of Nephrology and Hypertension, Center for Internal Medicine and Medical Clinic III, Klinikum Fulda, Fulda, Germany; 3 Institute of Pathology, University Medical Center, Göttingen, Germany; 4 Department of Medical Statistics, University Medical Center, Göttingen, Germany

**Keywords:** Sentinel lymph node, Melanoma, Nodal nevus, Skin nevi

## Abstract

**Purpose:**

Melanocytic nevi in lymph nodes (NNs) are an important histological differential diagnosis of initial sentinel lymph node (SN) metastasis in melanoma. Our aim was to associate NN in SNs with clinicopathologic features and survival rates in 1, 250 patients with SN biopsy for melanoma.

**Methods:**

To compare patients with present and absent NN, we used Fisher's exact test, Mann–Whitney *U* test, and multivariate logistic regression models in this retrospective observational study based on a prospectively maintained institutional database.

**Results:**

NN prevalence in axillary, cervical, and groin SNs was 16.5%, 19.4%, and 9.8%, respectively. NN were observed in combination with all growth patterns of melanoma, but more frequently when the primary was histologically associated with a cutaneous nevus. We observed a decreasing NN prevalence with increasing SN metastasis diameter. Multiple logistic regression determined a significantly increased NN probability for SNs of the neck or axilla, for individuals with ≥ 50 cutaneous nevi, midline primary melanomas, and for individuals who reported non-cutaneous malignancies in their parents. Cancer in parents was also significantly more frequently reported by melanoma patients who had more than 50 cutaneous nevi. In SN-negative patients, NN indicated a tendency for slightly lower melanoma-specific survival.

**Conclusions:**

We found a highly significant association between NN diagnosis and multiple cutaneous nevi and provided circumstantial evidence that cutaneous nevi in the drainage area of lymph nodes are particularly important. The trend toward lower melanoma-specific survival in SN-negative patients with NN suggests that careful differentiation of SN metastases is important.

## Introduction

Benign melanocytic nevus cell aggregates in lymph nodes, also called nodal nevi (NN), are typically located within the capsule or trabeculae of lymph nodes. NN were first depicted by Stewart and Copeland (1931). The described patient suffered from neurofibromatosis and a large bathing trunk nevus. Two different theories of NN histogenesis have been discussed, but none of them has gained general acceptance. Some authors consider NN to be remnants of embryonic migration of melanocyte precursors from the neural tube. The others favor a retrograde migration of nevus cells from the skin via afferent lymphatic vessels as causal.

Melanocytic nevi in sentinel lymph nodes (SNs) may cause diagnostic problems in patients with melanoma. An expert review of SN biopsy specimens found to be melanoma positive revealed that more than 10% were misclassified NN (El Sharouni et al. 2021). Misdiagnosis in the distinction between NN and SN metastases could result in over- or undertreatment. To date, the clinical significance of NN in SNs from patients with melanomas has rarely been investigated (Gambichler et al. 2013; de Beer et al. 2019; Yamashita et al. 2020). One objective of the present study was to correlate clinical and histological parameters with the presence of NN in SNs. In addition, our aim was to clarify the impact of the diagnosis of NN on the prognosis of sentinel-negative and sentinel-positive patients with melanomas.

## Methods

Our local ethics committee approved this retrospective observational study (No. 4/5/15), based on a prospectively maintained database of the Göttingen University Medical Center. We identified 1250 patients with primary cutaneous melanoma who underwent sentinel node biopsy (SNB) between April 1998 and December 2017. Surgical and pathologic standards were ensured throughout the study period by staff consistency. Indications for SNB were a Breslow thickness of ≥ 1 mm or < 1 mm if the Clark level was ≥ IV or if regression, ulceration or nodal tumor growth were documented. We excluded 23 patients in whom a SN could not be detected during surgery. Of the 1227 patients with successful SNB, 1085 had a documented histologic examination regarding the presence or absence of NN.

### Patient history and clinical examination

We used an a priori designed patient questionnaire and a standardized protocol for the first clinical examination at the time of SNB.

### SN mapping technique

Radioactive lymph nodes that appeared first during lymphoscintigraphy or displayed an afferent vessel were defined as SNs. During surgery, lymph nodes that stained blue or that emitted ≥ 10% of the radioactive signals of the most radioactive lymph node were defined as SNs (Kretschmer et al. 2015).

### Pathological SN assessment

We processed SNs using an extensive multiple slice protocol as previously described (Kretschmer et al. 2021). The reporting required indication on the presence or absence of an NN in each individual SN. H&E stains and the antibodies S100, Melan A, and HMB-45 were routinely employed. The immunohistochemical proliferation marker Ki-67 was used in difficult cases. NN were defined as monomorphous nests of melanocytes without cytological atypia, mitoses or prominent nucleoli, showing no or only weak HMB-45 reactivity. Histologic diagnosis was also based on the localization of melanocytes within capsule or trabeculae of the lymph node. Melanoma cells, nevus cells and pigmented histiocytes were meticulously differentiated based on anatomic localization, cytological and immunohistochemical criteria (Scolyer et al. 2008).

### Statistical analyses

The following medical history and clinical features were assessed for a relationship to NN prevalence: age, sex, history whether the melanoma arose from a pigmented mole, melanomas in parents or siblings, non-cutaneous malignancies in parents, Fitzpatrick skin phototype (I–IV in our sample), skin nevus count (< 50 vs. ≥ 50 skin nevi), multiple lentigines solares, histological features of the primary melanoma (location histological association with skin nevus, growth pattern, Breslow thickness, ulceration), location of the leading lymph node basin, unidirectional vs. bi- or multidirectional lymphatic drainage, SN status, and maximal SN metastasis diameter (MTD).

For judging correlations, Pearson’s correlation coefficient was used. To compare patients with NN present and NN absent, we used Fisher’s exact test for nominal and the Mann–Whitney *U* test for ordinal and metric variables. We further calculated odds ratios and their 95% confidence intervals (95% CI). We took variables having a p value of less than 0.2 in the univariate analysis into consideration for multivariate logistic regression models. Of predictors that showed strong association with each other (e.g., midline location of the primary melanoma and bidirectional lymphatic drainage), only the most appropriate one was included in multivariate analyses. Kaplan–Meier analyses evaluated follow-up time and survival rates according to the presence or absence of NN; the hazard ratio was calculated using Cox proportional hazards regression. Differences were compared with the log rank test. For statistical analyses, we used the statistical programming environment R (version 3.6.0; R Core Team 2018) and the statistical software package Statistica (Version 13.5 TIBCO Software). The significance level was set to α = 5%. Due to the exploratory nature of this study, no adjustment for multiple testing was applied.

### Follow-up

The patients were monitored routinely at 3-month intervals for the first 5 years and every 6 months for the next 5 years, in accordance with valid guidelines in Germany (Pflugfelder et al. 2013).

## Results

### Study population

The median follow-up time was 85 months (range 3–249 months). Of the 1085 patients with a valid examination for NN, 530 (48.8%) were female. The mean age was 58.5 ± 16.8 years, and the mean Breslow thickness was 2.4 ± 2.3 mm. Of the 1042 melanomas with an appropriate examination for ulceration, 291 (27.9%) were ulcerated.

At least one NN was documented within one of the SNs in 170 (15.7%) patients. The mean number of excised cervical, axillary and groin SNs was 2.9 ± 2.0, 2.0 ± 1.3, and 2.1 ± 1.1, respectively. The proportion of cases with bidirectional or multidirectional lymphatic drainage for neck, axilla, and groin was 35/135 (20.6%), 139/454 (23.4%), and 53/340 (13.5%), respectively. Thus, the frequency of multidirectional lymphatic drainage in patients with lymphatic drainage in the groin was significantly lower than for the cervical or axillary location of the leading lymph node area combined (*P* > 0.001). NN prevalence in axillary, cervical, and groin SNs was 16.5%, 19.4%, and 9.8%, respectively.

### Clinicopathological characteristics according to the presence or absence of NN

We observed NN in association with all histopathological growth patterns of primary melanoma. Specifically, NN prevalences according to growth pattern were: lentigo maligna melanoma 20% (9/45), superficial spreading melanoma 17.6% (73/415), nodular or superficial spreading melanoma with nodular component 14. 2% (66/471), acral lentiginous melanoma 7% (5/68), spitzoid melanoma 10% (3/30), desmoplastic melanoma 27.3% (3/8), nevoid melanoma 18.2% (2/13), malignant blue nevus 75% (3/4), and other rare growth patterns including melanoma of unclassifiable histogenetic type 30.8% (4/13). We compared other clinical and pathologic features according to the presence or absence of NN (Table 1). Regarding the leading nodal basin, NN prevalences in cervical and axillary SNs did not differ significantly (*P* = 0.40). SNs in the groin had a significantly lower prevalence. Accordingly, primary melanomas in the leg had a significantly lower NN probability than primary melanomas in the arm.

**Table 1:** Univariate comparisons: sentinel node nevus according to clinical and pathological characteristics

Feature	Level	No (valid observations)	No NN present (proportion)	Odds ratio	95% CI	*P*
Age	≤ 60 years	531	93 (17.5%)	0.76	0.54–1.07	0.17
	> 60 years	554	77 (13.9%)			
Gender	Female	530	79 (14.9%)	1.12	0.80–1.58	0.50
	Male	555	91 (16.4%)			
History that the melanoma arose from a pigmented mole	Yes	583	92 (15.8%)	1.00	0.62–1.51	1.0
	No	285	45 (15.8%)			
Melanoma in parents or siblings	Yes	75	15 (20.0%)	1.35	0.69–2.48	0.32
	No	959	150 (15.6%)			
Non-cutaneous malignancy in parents	Yes	282	59 (20.9%)	1.63	1.12–2.36	0.007
	No	708	99 (14.0%)			
Fitzpatrick skin phototype	Bright (1–2)	858	139 (16.2%)	1.15	0.73–1.84	0.54
	Dark (3–4)	201	29 (14.4%)			
Skin nevus count	< 50	648	70 (10.8%)	2.53	1.79–3.60	< 0.0001
	≥ 50	413	97 (23.5%)			
Multiple lentigines solares	Absent	471	69 (14.6%)	1.19	0.84–1.68	0.32
	Present	591	100 (16.9%)			
Primary melanoma location	Left body site	555	80 (14.4%)	0.99	0.68–1.44	0.98
	Right body site	440	63 (14.3%)			
	Lateralized	995	143 (14.4%)	2.68	1.58–4.45	< 0.0001
	Midline	87	27 (31.0%)			
	Extremities	541	65 (12.0)	1.850	1.28–2.69	< 0.0001
	Trunk	401	81 (20.2%)			
	Legs	317	23 (7.6%)	2.94	1.67–5.31	< 0.0001
	Arms	224	42 (18.8%)			
Melanoma with histologically associated skin nevus	No	271	52 (19.2%)	1.57	1.05–2.31	0.019
	Yes	684	90 (13.2%)			
Primary melanoma ulceration	Present	291	42 (14.4%)	1.09	0.74–1.65	0.64
	Absent	751	117 (15.6%)			
Leading nodal basin	Neck	158	26 (16.5%)	Groin vs other		0.0002
	Axilla	553	104 (19.4%)			
	Groin	369	36 (9.8%)	2.13	1.42–3.25	
Lymphatic drainage	Uni-directional	871	116 (13.2%)	2.17	1.47–3.17	< 0.0001
	Bi-directional	212	53 (25.0%)			
Pathological SN status	SN-negative	763	128 (16.7%)	1.34	0.91–2.01	0.12
	SN-positive	322	42 (13.0%)			
Maximal metastasis diameter	MTD > 1 mm	118	8 (6.9%)	2.74	1.19–7.12	0.01
	MTD ≤ 1 mm	204	34 (16.7%)			

*CI* confidence interval, *NN* nodal nevus, *SN* sentinel lymph node, *MTD* maximum diameter of the largest metastasis within the SNs

The NN rate was significantly higher in patients with multiple melanocytic nevi of the skin (≥ 50). SNs were significantly more frequently positive for NN, when they belonged to primary melanomas histologically associated with a melanocytic nevus.

We observed a significantly increased NN prevalence in patients who reported that one of their parents had a history of non-cutaneous malignancy.

Factors not significantly related to the presence of NN were, age, sex, the history of a mole preexisting the cutaneous melanoma, melanomas in relatives, Fitzpatrick skin phototype, the presence of multiple lentigines solares, Breslow thickness as a continuous variable, ulceration of the primary, and SN status.

In multiple logistic regression (Table 2), both the presence of more than 50 cutaneous nevi and nodal basins located in the upper half of the body were highly significant for increased NN probability. Midline-located primary tumors and history of non-cutaneous malignancies in parents also remained significant.

**Table 2:** Multivariate logistic regression analysis of factors predicting the probability of sentinel node nevi in the overall population

Feature	Odds ratio	95.0% CI	*P*
≥ 50 skin nevi	2.29	1.52–3.45	< 0.001
Nodal basin other than groin	2.30	1.44–3.68	< 0.001
Midline location of the primary melanoma	0.49	0.27–0.89	0.018
Parental non-skin malignancy	1.58	1.05–2.37	0.029
Pathological SN status	1.34	0.85–2.13	0.205
Melanoma with histologically associated skin nevus	1.15	0.75–1.77	0.514
Age/year	0.99	0.98–1.01	0.59

*CI* confidence interval, *SN* sentinel lymph node,

#### Association of multiple cutaneous nevi with the history of non-cutaneous cancer in parents

To further explain the surprising association between NN and family history of parental cancer, we also examined the association with multiple skin nevi. Patients with cutaneous high-risk melanomas who had multiple nevi of the skin reported significantly more often non-cutaneous cancer in parents (20.9% vs. 14.0%, *P* < 0.001).

## Peculiarities according to SN status

### SN-negative subpopulation

In the SN-negative subpopulation, the same factors were significant for NN that have already been described for the overall population prevalence (detailed results not shown). In contrast to the overall population, multiple solar lentigines also indicated a higher rate of NN (19.6% vs. 13.7%, *P* = 0.039). The histologic association of a primary melanoma with a melanocytic nevus failed to reach significance to predict the NN rate (20.1% vs. 14.6%, *P* = 0.083).

In multivariate logistic regression (Table 3), the presence of more than 50 skin nevi and SNs draining the upper half of the body remained significant.

**Table 3:** Multivariate logistic regression analysis of factors predicting the probability of nodal nevi according to the pathological status of the sentinel lymph node

Feature	SN status negative			SN status positive		
	Odds ratio	95% CI	*P*	Odds ratio	95% CI	*P*
≥ 50 skin nevi	2.22	1.43–3.57	< 0.001	3.68	1.66–8.06	0.001
SN from the neck or axilla (vs. groin)	2.52	1.43–4.48	0.001	1.84	0.85–4.03	0.120
Non-cutaneous malignancy in parents	1.39	0.86–2.24	0.178	2.24	1.03–4.89	0.041
Midline location of the primary melanoma	1.82	0.93–3.58	0.082	2.77	0.91–8.46	0.073
Age / year	0.99	0.98–1.01	0.596	1.004	0.98–1.03	0.710
Multiple solar lentigines	1.09	0.69–1.72	0.725	0.43	1.99–0.94	0.034
MTD / mm	–	–	–	0.69	0.48–0.99	0.043

*CI* confidence interval, *SN* sentinel lymph node, *MTD* maximum diameter of the largest metastasis within the SN

#### SN-positive subpopulation

Also in the presence of SN metastasis, in univariate analysis NN were associated with midline-located primary melanomas (30.4% vs. 15.0%, *P* = 0.019), bidirectional lymphatic drainage (24.6% vs. 9.9%, *P* = 0.002), and skin nevus count of ≥ 50 (22.4% vs. 7.3%, *P* < 0.001). The history of life-threatening cancer in parents failed significance (NN rates 19.9% vs 11.2%, *P* = 0.18). In contrast to SN-negative patients, Breslow thickness (*P* = 0.005) as continuous variable turned out to be important. Breslow thickness was correlated with the MTD (*r* = 0.39, *P* < 0001). A maximum metastasis diameter exceeding 1 mm was associated with a decreased probability of NN detection (18.5% vs. 6.2%, *P* = 0.010) (Tables 1, 3). Other features were non-significant.

In multivariate logistic regression (Table 3), the presence of more than 50 skin nevi, SNs draining the upper half of the body, and history of parental non-cutaneous cancers remained significant also in SN-positive patients.

### Survival analysis

**Fig. 1:**
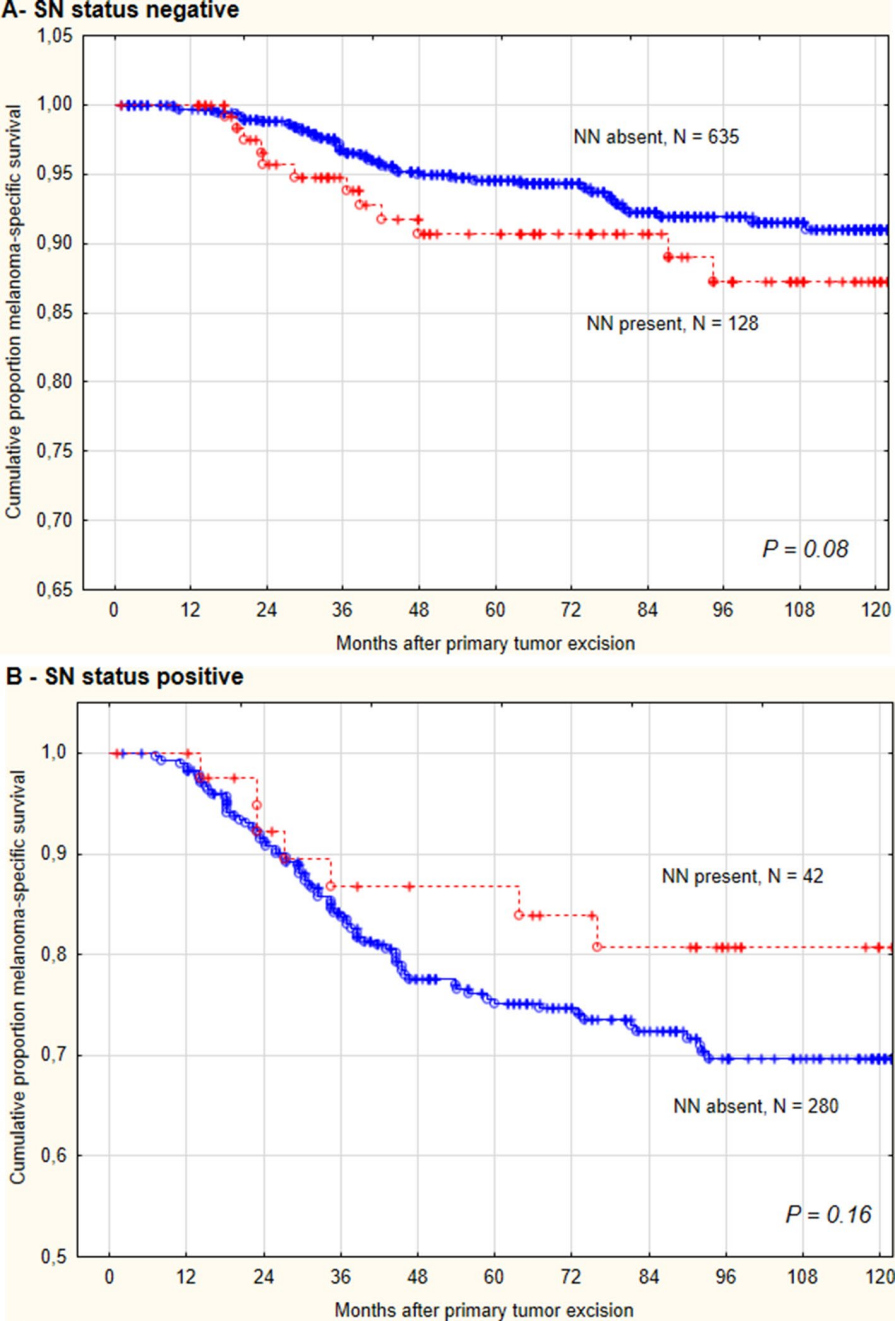
Melanoma-specific survival according to sentinel node (SN) status. **A** SN-negative patients with intranodal nevus (NN) show a slightly decreased survival rate. **B** SN-positive patients with the simultaneous diagnosis of NN and nodal metastasis fared slightly better than patients with SN metastasis only. The differences in the survival curves failed significance

SN-negative patients had approximately 4% decreased melanoma-specific 5-year survival when diagnosed with NN (hazard ratio 1.7 for NN present; 95% CI 0.93–3.13, log-rank test: *P* = 0.082, Fig. 1A. Primary tumor-related risk factors did not differ significantly between the subgroups with and without NN (median Breslow thickness 1.4 mm for both (range 0.4–20.0 mm), *P* = 0.650, ulceration 24.4% vs. 23%, *P* = 0.722). There were no significant differences in recurrence-free survival (*P* = 0.32) and recurrence-free survival in the nodal basin (*P* = 0.15).

SN-positive patients with NN had slightly better melanoma-specific survival than patients without NN (Fig. 1B). The difference in survival curves was not significant. It should be noted that SN-positive patients with concurrent NN had a lower median primary tumor thickness than patients with only SN metastases (median Breslow thickness 1.6 mm (range 0.65–5.2 mm) vs. 2.4 mm (range 0.4–15.0 mm), P = 0.005) and also a lower median MTD (0.80 mm (range 0.02–5.0 mm) vs. 1.6 mm (range 0.002–14.0 mm), *P* < 0.001).

## Discussion

**Fig. 2:**
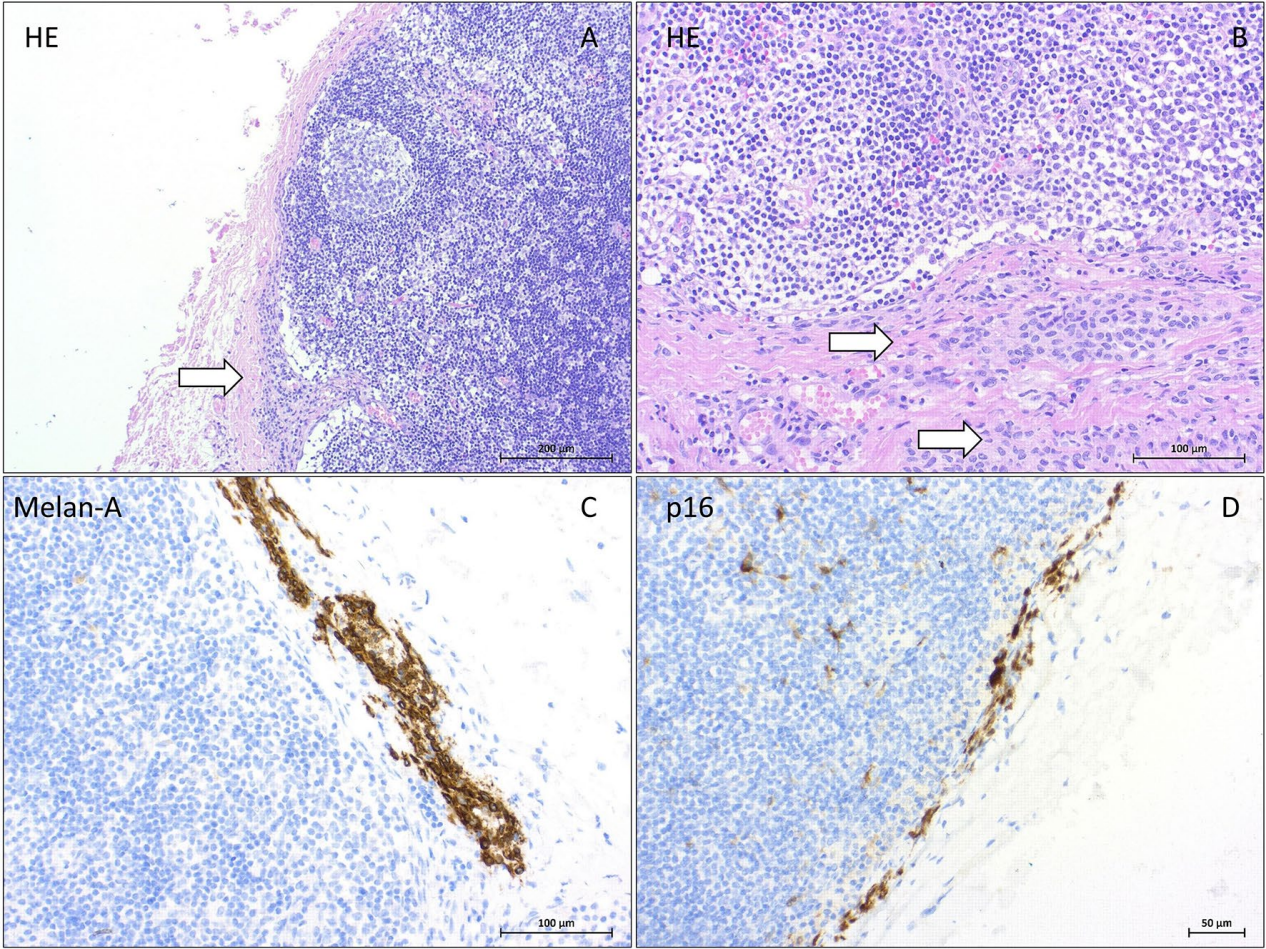
Histology and immunohistochemistry of melanocytic nevi in a sentinel lymph nodes: In the H&E staining, small groups of melanocytic cells in the nodal capsule (⇒) show bland cytomorphology. Localization of melanocytes within the capsule or trabeculae of the lymph node strongly supports the diagnosis of a benign NN (**A** HE × 100; **B** HE × 200). Positive cytoplasmatic staining for Melan A (**C **× 200) and positive nuclear staining for p16 (**D **× 200)

NN are growth-arrested melanocytic clusters that are usually diagnosed in SNs draining melanoma, breast, vulvar, or penile cancer. NN and other benign inclusions in lymph nodes are addressed in a recent review article (Müller et al. 2021). Unlike most melanomas, NN usually have bland cytomorphology and no mitotic activity. With the exception of intranodal blue nevi, NN do not express HMB-45 or express it only weakly (Gonzàlez-Farré et al. 2020). Histological images of NN are shown in Fig. 2. Recently, p16 (mostly positive for NN, Fig. 2) (Piana et al. 2015) and PRAME (mostly negative for NN) (See et al. 2020) have been introduced in the differential diagnosis to SN metastases of melanoma.

The NN prevalence reported in the literature vary considerably, from 0 to 22% (McCarthy et al. 1974; Fisher et al. 1994; Biddle et al. 2003; Holt et al. 2004; Gambichler et al. 2013; Piana et al. 2015; Smith et al. 2016; Kim et al. 2018; de Beer et al. 2019; Yamashita et al. 2020) The likelihood of an NN diagnosis is certainly influenced by many factors, including the type of cancer, the pathologic protocol used, the lymphatic drainage conditions, the size of the lymph nodes, the number of lymph nodes removed, and the frequency and extent of malignant metastases. Using an extensive pathologic protocol, we observed an overall prevalence of 15.7% in a large population with SNB for cutaneous melanomas. The specific prevalences for cervical, axillary, and groin SNs were 16.5%, 19.4%, and 9.8%, respectively.

Regarding etiology, it has been speculated that during embryogenesis NN develop from melanocytic precursor cells that migrate from the neural crest to the skin, taking the dorsolateral developmental pathway (Kos et al. 2001). Melanocytic precursors migrate through the mesenchyme, where the lymphatic system is formed almost simultaneously. It has been argued that the location of NN predominantly in fibrous structures of lymph nodes, the association of NN with congenital nevi (Bowen et al. 2015), and the mere existence of neurocutaneous melanosis would support the theory of cell arrest during embryogenesis (Carson et al. 1996).

On the other hand, there are a number of good arguments that NN stem from melanocytic cells of the skin that have migrated via afferent lymphatic vessels: (1) In agreement with Carson et al. (1996), at least in univariate analysis, we documented a significantly increased probability of NN in patients whose primary melanomas were histologically associated with a cutaneous nevus. (2) Invasion of lymphatic vessels by nevus cells has been observed within cutaneous nevi (McCarthy et al. 1974; Bell et al. 1979; Subramony and Lewin 1985; Hara 1993). (3) Cutaneous nevi in the drainage area of lymph nodes with NN have been observed in several case series (McCarthy et al. 1974; Hara 1993; Fontaine et al. 2002; Holt et al. 2004; Hu et al. 2020) (4) NN have been much more frequent in SNs than in non-SNs (Carson et al. 1996; Holt et al. 2004; Gambichler et al. 2013) or lymphadenectomy specimens.(Ridolfi et al. 1977; Fisher et al. 1994) (5) NN have not been described in deeply located nodes of the thorax or abdomen, which do not drain the skin. (McCarthy et al. 1974) (6) Lymph nodes from melanoma surgery are more likely to contain NN than superficial lymph nodes excised for other cancers (Biddle et al. 2003; Piana et al. 2015) (7) electron microscopic studies of NN have revealed ultrastructural features identical to those of intradermal nevus cells (Erlandson and Rosen 1982). (8) Using next-generation sequencing, comparison of mutation profiles in primary melanomas and corresponding NN suggested that NN descend from cutaneous melanocytes, rather than from primary MM or arrested progenitor cells (Gambichler et al. 2021).

We demonstrated that NN can be found in SNs related to all growth patterns of primary melanoma. This includes rare growth patterns such as desmoplastic and nevoid melanomas. The SNs of three out of four patients with malignant blue nevus displayed an NN.

Using multivariate analyses, we discovered a very robust association between multiple nevi of the skin and the prevalence of NN, both in SN-negative and SN-positive patients. To the best of our knowledge, only 1 small case–control study including 22 patients with NN has confirmed this observation (Ribero et al. 2017). We found that the midline location of the primary melanoma as well as bidirectional lymphatic drainage resulted in a higher number of excised SNs, which logically increases the chance of detecting NN. As many as 31% of patients with upper trunk melanoma had bidirectional lymphatic drainage (Kretschmer et al. 2019). A lower NN prevalence in inguinal lymph nodes has already been reported (McCarthy et al. 1974; Gambichler et al. 2013). By reporting prevalence for cervical SNs for the first time, we can state a significantly higher NN prevalence for SNs draining the upper part of the body, which hold true in multivariate logistic regression. This coincides with the distribution of cutaneous nevi. Also their density seems to be higher on the upper half of the body (Echeverría et al. 2014). Many studies have shown an association between sun exposure and the number of skin nevi. Cervical lymph nodes drain a relatively small area of skin that is chronically exposed to sunlight. Axillary lymph nodes drain large areas of intermittently sun-exposed skin, which is characterized by high skin nevus count (Newton-Bishop et al. 2010). In a longitudinal study, an increase in skin nevi after 15 years of observation was registered only on the upper parts of the body.(Ribero et al. 2021) We confirmed that primary melanomas located at the arm were associated with higher NN rates than leg-located melanomas (Yamashita et al. 2020). It has been shown that also the density of cutaneous nevi of the arms is greater than that of nevi of the legs (Harrison et al. 1999). Interestingly, the frequency of ultraviolet of ultraviolet light-associated mutations was relatively high not only in primary melanomas but also in NN (Gambichler et al. 2021). Moreover, ultraviolet light seems to induce the expression of growth factors involved in the early migration process of malignant melanocytes (Wäster et al. 2017).

Like others, we found no sex-specific difference in the NN rates. Fitzpatrick skin phototype was not associated with NN, an observation not previously reported. The influence of multiple solar lentigines was not convincing because it had different signs depending on SN status (Table 3).

We can only speculate about the possible implications of our surprising observation that patients with melanoma in whom at least one parent had undergone another life-threatening cancer were significantly more likely to be diagnosed with NN. In parallel, multiple nevi of the skin were also highly significantly associated with a history of cancer in the parents. Genome-wide association studies have described a larger number of susceptibility loci for melanoma that are related not only to nevus count, pigment type, and tanning response but also to telomere maintenance and DNA repair mechanisms. From this perspective, a link between multiple melanocytic nevi in skin and lymph nodes and a susceptibility to develop other tumor entities seems potentially explainable (Landi et al. 2020).

Analyzing 56 SN-negative patients with NN, Yamashita et al. observed a non-significantly lower recurrence-rate, compared with purely SN-negatives and suggested a metastasis-protective effect of NN (Yamashita et al. 2020). Most previous studies (Gambichler et al. 2013; Smith et al. 2016; Kim et al. 2018; de Beer et al. 2019; El Sharouni et al. 2021) have concluded that the presence of NN in SN-negative cases does not affect survival. We can generally confirm this statement but found a trend toward slightly higher mortality in SN-negatives with NN (Fig. 1A). A large Dutch registry study of 11,274 patients confirmed a similar trend. (de Beer et al. 2019) Possibly, in daily practice SN metastases are misinterpreted as NN in rare cases. For example, metastases of nevoid melanomas can strongly resemble NN (Biddle et al. 2003; Davis et al. 2016). Very small metastases, metastases in intracapsular lymphatic vessels, NN in the presence of concurrent SN metastases, and lack of HMB-45 reactivity of SN metastases represent further diagnostic pitfalls (Biddle et al. 2003; Gonzàlez-Farré et al. 2020; Lezcano et al. 2020). Very rarely, metastases of other kinds of cancer, e. g., lobular breast cancer may mimic NN (Fisher et al. 1994).

Prevalence and prognostic impact of NN diagnosis in SN-positive patients have not been reported. In our study, patients who were both SN and NN positive had a slightly higher survival rate than SN-positive patients without NN. We demonstrated that the likelihood of an NN diagnosis decreases with increasing Breslow thickness and, relatedly, with increasing SN metastasis size. This alone explains why the SN-positive patients with concurrent NN diagnosis fared somewhat better (Fig. 1B).

Our study has several limitations, including retrospective data analysis, the failure to record the number of SNs affected with NN per subject, the microanatomical location of NN within SNs, and the histologic specificities of NN according to the growth patterns of primary melanoma. Multiple testing is another problem; we cannot exclude the possibility that some error probabilities are due to chance.

In summary, using multivariate analyses, we found a highly significantly increased prevalence of NN in SNs in patients with upper body melanomas, midline primary melanomas, and those with more than 50 nevi of the skin. A history of non-cutaneous malignancies in parents was associated with increased NN prevalence, as were multiple nevi of the skin. When the primary melanoma was histologically associated with a cutaneous nevus, NNs were also more frequently found in SNs. In SN-positive patients, the likelihood of an NN diagnosis decreased with increasing metastatic diameter. The slightly worse melanoma-specific survival of SN-negative patients with NN that we observed suggests that careful differentiation of NN and SN metastases is sometimes difficult and should be done very carefully.

## Abbreviations

MTD: Maximum diameter of the largest intranodal metastasis
NN: Melanocytic nevi in (sentinel) lymph nodes
SN: Sentinel lymph node
SNB: Sentinel lymph node biopsy
vs: Versus
